# Unifying Repbase and Dfam: a new open foundation for transposable element research

**DOI:** 10.1186/s13100-026-00409-9

**Published:** 2026-06-25

**Authors:** Kenji K. Kojima, Arian F. A. Smit, Weidong Bao, Oleksiy Kohany, Noriko F. Kojima, Timothy Jurka, Robert Hubley, Travis J. Wheeler

**Affiliations:** 1 Genetic Information Research Institute, East Palo Alto, 94303 CA USA; 2 Institute for Systems Biology, Seattle, 98109 WA USA; 3 College of Pharmacy, University of Arizona, Tucson, 85719 AZ USA

**Keywords:** Transposable elements, Repository, Integration

## Abstract

Repbase and Dfam, the two foundational resources for transposable element annotation, are being unified into a single, fully open access framework, with Repbase released under CC-0 and its core curation team joining the Dfam project.

Accurate identification and classification of transposable elements (TEs) and their remnants is foundational to genome assembly, gene annotation, evolutionary analysis, and an expanding range of biomedical and agricultural applications. For over three decades, this work has depended on two complementary resources: Repbase [1, 2] and Dfam [3, 4]. We write to announce that these resources are being brought together under a unified, fully open access framework (Fig. 1).

**Fig. 1:**
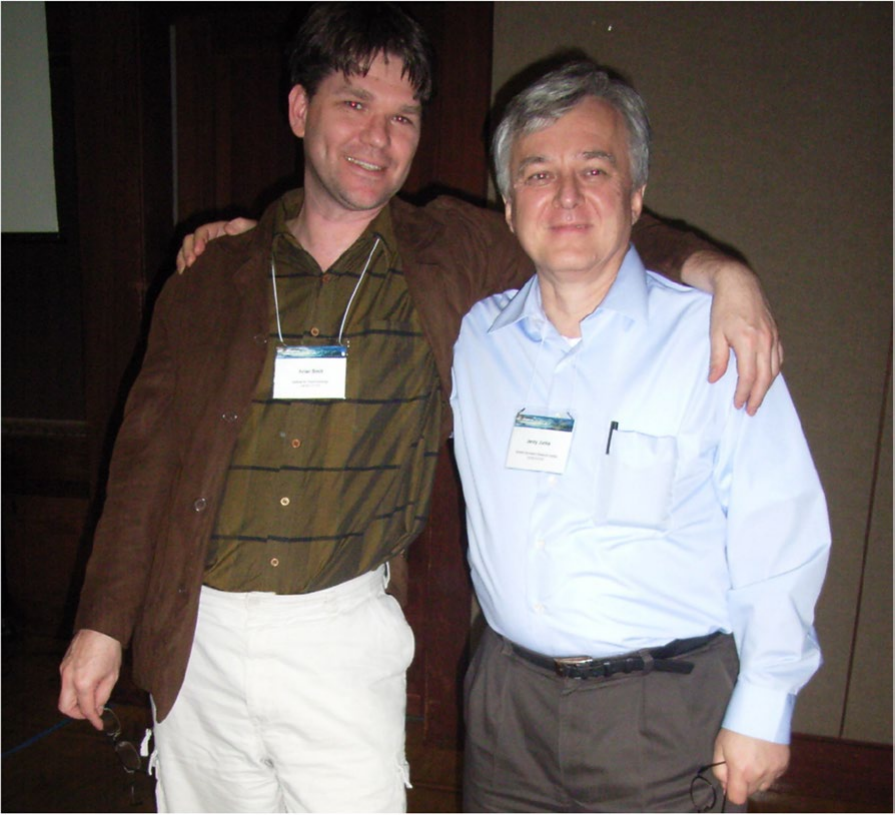
Arian Smit (left) and Jerzy Jurka (right) at the 2006 Genomic Impact of Eukaryotic Transposable Elements conference at Asilomar. Twenty years later, Repbase and Dfam are coming together. (photo by Jerzy Kulski)

Repbase was founded in 1990 by Jerzy Jurka at the Linus Pauling Institute of Science and Medicine and relocated to the Genetic Information Research Institute (GIRI) in 1994, where it has served as the primary reference collection of eukaryotic TE consensus sequences for the genomics community. Arian Smit was an earlier contributor to Repbase and his RepeatMasker software became the library’s most prominent user and its main pathway to the broader community. The strength of Repbase lies in meticulous expert curation: each entry reflects careful attention to family boundaries, classification, and consensus reconstruction. Over the years, the team at GIRI, with contributions from the community, expanded the database to cover TEs across a wide range of eukaryotic lineages, characterizing numerous superfamilies and contributing foundational knowledge to the field.

Dfam originated in 2012 as an alternative and open resource. It represents TE families with profile hidden Markov models (HMMs) built from seed alignments of genomic instances; because the underlying instances and their genomic coordinates are retained, the evidence supporting each family model remains transparent and traceable. Developed by a broad collaboration that included Jerzy Jurka, it is now maintained by Robert Hubley and Arian Smit at the Institute for Systems Biology, and Travis Wheeler at the University of Arizona. Dfam was designed from the outset as an open access resource. It has since grown to house TE family models for nearly 3,000 species, supported by partnerships with the European Bioinformatics Institute, the Vertebrate Genomes Project [5], the Zoonomia project [6], and other large-scale sequencing efforts. Dfam now serves as the default open TE library for RepeatMasker.

While both resources have been indispensable, they have evolved along separate paths. Repbase offers unmatched depth of expert curation but has operated under a restrictive licensing model and subscription fees were introduced for all users in 2019 due to widespread cuts in NIH funding for life sciences databases. Dfam provides open access and scalable infrastructure but has absorbed large volumes of de novo generated libraries that have not undergone the level of manual refinement characteristic of Repbase entries. The community has long needed both the curation depth of one and the openness and scalability of the other.

In the 2016 Dfam paper [7], reflecting a growing relationship between the two projects, we wrote that “in the coming years we will... continue the protocol used here to build alignments and profile HMMs [based on] the Repbase-derived RepeatMasker library”. A decade later, we are pleased to announce a far more comprehensive realization of that goal: GIRI has decided to release the full Repbase collection under a CC-0 (public domain) license and to partner with the Dfam team to integrate these important resources. Concurrent with this transition, the core Repbase group will soon be joining the Dfam team. There, they will continue their expert curation work and play a key role in efforts to merge the two resources. To catalyze this transition, GIRI is providing a generous gift to the University of Arizona that will seed the personnel and operational work required to begin building the integrated resource.

The integration effort is significant. It will require developing methods to faithfully convert curated Repbase consensus sequences into Dfam’s seed alignment format, reconciling overlapping and redundant family entries across the two databases, and migrating GIRI’s specialized curation tools and workflows into the Dfam infrastructure. The integration effort will proceed in stages over the coming releases. An initial open release of Repbase data will be made available in its current form, followed by progressive incorporation into the Dfam framework.

Beyond the immediate data and software integration, we plan to leverage the combined expertise to develop improved methods for semi-automated curation of the rapidly growing volume of de novo TE libraries being contributed by the community. We also plan to expand community engagement through workshops, tutorials, and enhanced web services that preserve features valued by Repbase users while taking advantage of Dfam’s open platform.

We view this as a pivotal moment for the field. Decades of expert knowledge accumulated at GIRI will now be channeled into a fully open resource, available to every researcher without restriction. This unified effort will benefit researchers at every scale, whether annotating a single newly sequenced genome or generating TE libraries across hundreds of species. The result will be a single, coherent resource that combines Repbase’s curation depth with Dfam’s open infrastructure and broad taxonomic coverage. The new collaborative team is excited to work with the TE research community to build on this foundation.
